# Correlation in the change of gut microbiota with clinical periodontal parameters in grade C periodontitis patients after non-surgical periodontal therapy

**DOI:** 10.1099/jmm.0.002065

**Published:** 2025-10-09

**Authors:** Elif Mutafcilar Velioglu, Uğur Arslan, Seyit Ali Kayis, Salih Maçin, Nobuhiko Kamada, Sema S. Hakki

**Affiliations:** 1 Department of Periodontology, Faculty of Dentistry, Selcuk University, Konya, Turkey; 2 Department of Microbiology, Faculty of Medicine, Selcuk University, Konya, Turkey; 3 Department of Biostatistics, Faculty of Medicine, Bolu Abant Izzet Baysal University, Bolu, Turkey; 4 Department of Internal Medicine, Division of Gastroenterology, University of Michigan Medical School, Ann Arbor, MI, USA

**Keywords:** gut microbiota, metagenomics, oral microbiota, periodontal health, periodontitis

## Abstract

A corrigendum of this article has been published full details can be found at https://doi.org/10.1099/jmm.0.002120

**Introduction.** Intestinal dysbiosis is associated with systemic health, and approaches targeting the microbiome can influence the host. Oral and intestinal microbiota are interrelated; therefore, we aimed to determine whether non-surgical periodontal treatment (NSPT) affects systemic health through its impact on the intestinal microbiota.

**Hypothesis/Gap Statement.** Although the association between oral and gut microbiota has been suggested, there is limited evidence regarding how periodontal therapy may influence intestinal microbial composition. We hypothesized that NSPT in patients with periodontitis would lead to favourable changes in the gut microbiome, which may parallel improvements in clinical periodontal parameters.

**Aim.** This study aimed to investigate the effect of NSPT on both oral and intestinal microbiota and to evaluate whether changes in gut microbial composition correlate with periodontal clinical outcomes.

**Methodology.** Five systemically healthy individuals with grade C periodontitis and five systemically and periodontally healthy individuals were included. Saliva and stool samples were collected at baseline and 1 month after NSPT. DNA extractions were performed and subjected to 16S ribosomal RNA gene sequencing on the Illumina Novaseq at the V3–V4 hypervariable regions.

**Results.** Grade C periodontitis patients displayed distinct oral and gut microbiomes compared to healthy individuals. NSPT resulted in a reduction in the diversity of both saliva and stool samples in healthy individuals (*P*>0.05). Salivary *Fusobacteriota* levels (*P*<0.05) and the gut *Firmicutes*/*Bacteroides* ratio decreased after NSPT. Moreover, changes in gut microbiota significantly correlated with improvements in periodontal probing depth and clinical attachment level in periodontitis patients.

**Conclusion.** The improvement in clinical periodontal parameters after NSPT correlates with a positive shift in the gut microbiome towards health. Although the number of participants was limited, these findings support a strong relationship between periodontal and gut status. Further studies with larger cohorts and long-term follow-up are required to confirm these results.

**Impact Statement**

**Scientific rationale for the study**

The balance of the bacterial composition of intestinal microflora is associated with the holistic state of well-being. Targeting intestinal dysbiosis may significantly benefit treating periodontitis as a chronic inflammatory systemic disease.

**Principal findings**

Metagenomic analyses revealed that both oral and gut diversity changed towards the healthy profile after non-surgical periodontal treatment.

**Practical implications**

The effect of periodontal therapy on gut microbiota and chronic systemic inflammatory diseases may be seen more clearly with further studies and contribute to treatment approaches for both periodontal and systemic diseases.

## Introduction

Numerous studies have reported the relationship between systemic diseases/conditions and oral health and the positive additive effects of periodontal therapy on systemic conditions. However, there is insufficient evidence regarding the interaction of oral microbiota and gut health. The gastrointestinal tract starts with the oral cavity and ends with the gut. Oral micro-organisms translocate to the gut and then colonize in the intestine. The oral cavity, including oral mucosa, teeth surfaces, periodontal pocket and tongue dorsum, is considered a reservoir for gut microbiota. The change in oral microbiota might affect the gut’s health.

The negative shift of colonizers and dysbiosis of oral microbiota, especially supra- and subgingival microorganisms, leads to gingival inflammation, ulceration of the gingival sulcus, gingival bleeding, periodontal pocket and attachment loss. Periodontal disease progresses due to the increased colonization of pathogenic microorganisms and their interactions, leading to an increase in periodontal pocket depths. The increase in periodontal pocket depth further complicates these interactions, resulting in dysbiosis and an exacerbation of periodontal disease severity. Progression of the periodontal disease results in chronic inflammation and loss of periodontal tissue, including periodontal ligament and alveolar bone. Gingival recessions can also occur.

Chronic inflammation in the periodontal environment contributes to systemic inflammation. This systemic inflammation participates in the aetiology of other inflammatory diseases and makes patients non-responsive to systemic medical therapies. The therapeutic prognosis of the patient was affected negatively by periodontal diseases if they remained untreated. There is a definite relationship between the treatment of periodontal disease and the better response of patients with systemic diseases, including diabetes mellitus, cardiovascular diseases, Alzheimer’s and multiple sclerosis to their systemic treatments [1]. So, we may speculate that the treatment of periodontal diseases will positively affect gut diseases.

Recently, Imai *et al*. displayed a potential pathogenic association between periodontal disease and Crohn’s disease. In murine models, Kitamoto and Kamada reported a complex intermucosal connection between the mouth and the gut. Orally primed pathogenic T cells can transmigrate to the gut, and ingested oral pathobionts can reactivate them in the gastrointestinal tract, then exacerbate intestinal inflammation and can be a risk for developing or relapsing intestinal bowel diseases [2]. Chen *et al*. reported that oral–gut transmission of microbes contributed to hypertension and suggested that regular monitoring of periodontal disease and targeting oral–gut microbial transmission may become effective strategies to improve the prevention and treatment of hypertension [3].

The cycle among oral microbiota, gut microbiota and systemic diseases proposes that dysbiosis of oral microbiota is the crucial point for future treatment modalities, and this perspective will change the guidelines of the treatments of chronic inflammatory diseases. Evidence regarding the correlation between the change in clinical periodontal parameters and the change in gut microbiota is critical for future approaches. This study aims to explore whether there is dysbiosis in the gut of periodontitis patients and to determine whether non-surgical periodontal therapy reverses gut dysbiosis of periodontitis patients.

## Methods

### Study design and clinical procedures

The study group comprised five individuals with grade C stage III periodontitis (2 men and 3 women, age: 30.2±9.62). The control group consisted of systemically and periodontally healthy participants (2 men and 3 women, age: 28.6±1.52). All patients were informed about the purposes, treatment procedures and duration of the present study, and written informed consent was obtained from all participants. The design of the study can be seen in Fig. S1, available in the online Supplementary Material.

The baseline evaluation included the calculation of body mass index (BMI) using the ratio of weight to the square of height (kg m^−^²) and a comprehensive full-mouth periodontal examination utilizing a periodontal probe (HuFriedy, Chicago, IL, USA). This examination assessed plaque index (PI), which reflects oral hygiene; gingival index (GI) and bleeding on probing (BOP), which indicate gingival inflammation; and periodontal pocket depth (PPD) and clinical attachment level (CAL), which quantify the extent of periodontal destruction [4]. Medical and dental history was obtained and reviewed along with exclusion criteria before the periodontal examination. Patients were excluded if they used any antibiotics for the last 6 months, received immunosuppressive drugs, had periodontal therapy for the last 1 year or had any other systemic diseases. Smokers, alcohol consumers and any status that affects hormonal circulation were also excluded.

Healthy (H) individuals were chosen based on a PD ≤3 mm and no gingival inflammation (no gingival redness/oedema), no more than 10% of the sites with bleeding on probing and no radiological evidence for crestal alveolar bone loss according to the 2017 Classification of Periodontal and Peri-Implant Diseases and Conditions [5]. Radiographic records can be seen in Fig. S2. Demographic findings of the healthy and grade C periodontitis patients are presented in Table 1.

**Table 1.** Demographic findings of the healthy and grade C periodontitis patients

Groups	Periodontitis (*n*: 5)			Control (*n*: 5)	*P*
Periodontal status		Grade C stage III		Periodontal health	
Gender (F/M)		3/2		3/2	*P*>0.05
Age (ave ±dev)		30.2±9.62		28.6±1.52	*P*>0.05
BMI (ave ±dev)		22.5±8.14		20.92±4.81	*P*>0.05
Smoking		No		No	
	**Baseline**		**After NSPT (1 month)**		
PPD (ave ±dev)	3.89±0.36^a^		3.188±0.67^a^	1.96±0.22^b^	*P*<0.05
CAL (ave ±dev)	3.98±0.35^a^		3.318±0.69^a^	0.182±0.04^b^	*P*<0.05
GI (ave ±dev)	1.8±0.117^a^		1.4±0.11^b^	1.12±0.1^c^	*P*<0.05
BOP (ave ±dev)	0.76±0.006^a^		0.42±0.09^b^	0.076±0.023^c^	*P*<0.05
PI (ave ±dev)	1.923±0.39^a^		1.3±0.07^a^	1.12±0.1^b^	*P*<0.05

### Periodontal therapy

As a treatment for the study group, oral hygiene instructions were given to all individuals during the first visit. Supragingival scaling was performed by hand and ultrasonic instruments. Non-surgical periodontal therapy (NSPT) as full-mouth subgingival SRP under local anaesthesia was completed in a single visit (~80–90 min) with an ultrasonic device (Satelec) and hand instruments (Gracey Curettes, HuFriedy) without any antibiotic regimen and antimicrobial irrigation/mouthwash.

### Saliva and stool sample collection

Clinical examinations and saliva/stool sampling were performed at baseline and post-therapy (1 month after treatment) by the same calibrated investigator (EEMV). Saliva and stool samples were collected from groups before clinical evaluation. Saliva samples were collected into plastic cups for 5 min and then transferred to plastic tubes. Then, the total saliva collected was aspirated from a disposable 5-ml sterile syringe. Stool samples were collected in sterile recipients previously provided, and samples were stored at −80 °C until metagenomic analysis.

### Sample processing and 16S rRNA gene sequencing

A commercial DNA extraction kit (DNeasy Blood and Tissue Kit, QIAGEN, Hilden, Germany) was used for bacterial DNA extraction from saliva and stool samples considering the manufacturer’s instructions, 16S rRNA genes of V3–V4 regions were amplified using a specific primer (515 F-806R) and PCR reactions were carried out with Phusion High-Fidelity PCR Master Mix (New England Biolabs). The mixed PCR products were purified with a QIAGEN Gel Extraction Kit (QIAGEN, Germany). The libraries were generated with NEBNext UltraTM DNA Library Prep Kit and quantified via Qubit and quantitative polymerase chain reaction (qPCR) to be analysed by the Illumina platform. Paired-end reads were assigned to samples based on their unique barcodes and truncated by cutting off the barcode and primer sequences. Paired-end reads were merged using flash (v. 1.2.7), an analysis tool designed to merge paired-end reads when reads generated from opposite ends of the same DNA fragment overlap [6]. Quality filtering on the raw tags was performed to obtain high-quality, clean tags according to qiime (v. 1.7.0) [7,8]. After chimaera sequences were removed, effective tags were obtained [9]. To study the microbial community composition in each sample, operational taxonomic units were obtained by clustering with 97% identity on the effective tags [10]. Alpha diversity is applied in analysing biodiversity for a sample through five indices, including observed species, Chao1, Shannon, ACE and Good’s coverage. All these indices in our samples were calculated with qiime (v. 1.7.0) and displayed with R software (v. 2.15.3).

### Statistical analysis

Clinical periodontal parameters of periodontitis before and after treatment and those of the healthy group were compared using one-way ANOVA and Tukey test as a post hoc test. The relationship between clinical and microbiome variable changes at pre- and post-treatment is evaluated using the Pearson moment product correlation. All statistical analysis was performed using R version 3.6.0 (The R Foundation for Statistical Computing, Vienna, Austria, https://www.r-project.org). Shapiro–Wilk’s normality test and Q–Q plots were used to check the normality of the data. Levene’s test was also used to assess the homogeneity of the variance. Numerical data were presented as mean±sd. A Mann–Whitney *U* test was run to determine whether there was a statistically significant difference between study groups. A two-tailed *P*-value of less than 5% was considered statistically significant.

## Results

### Clinical outcomes after NPT

**Fig. 1.**
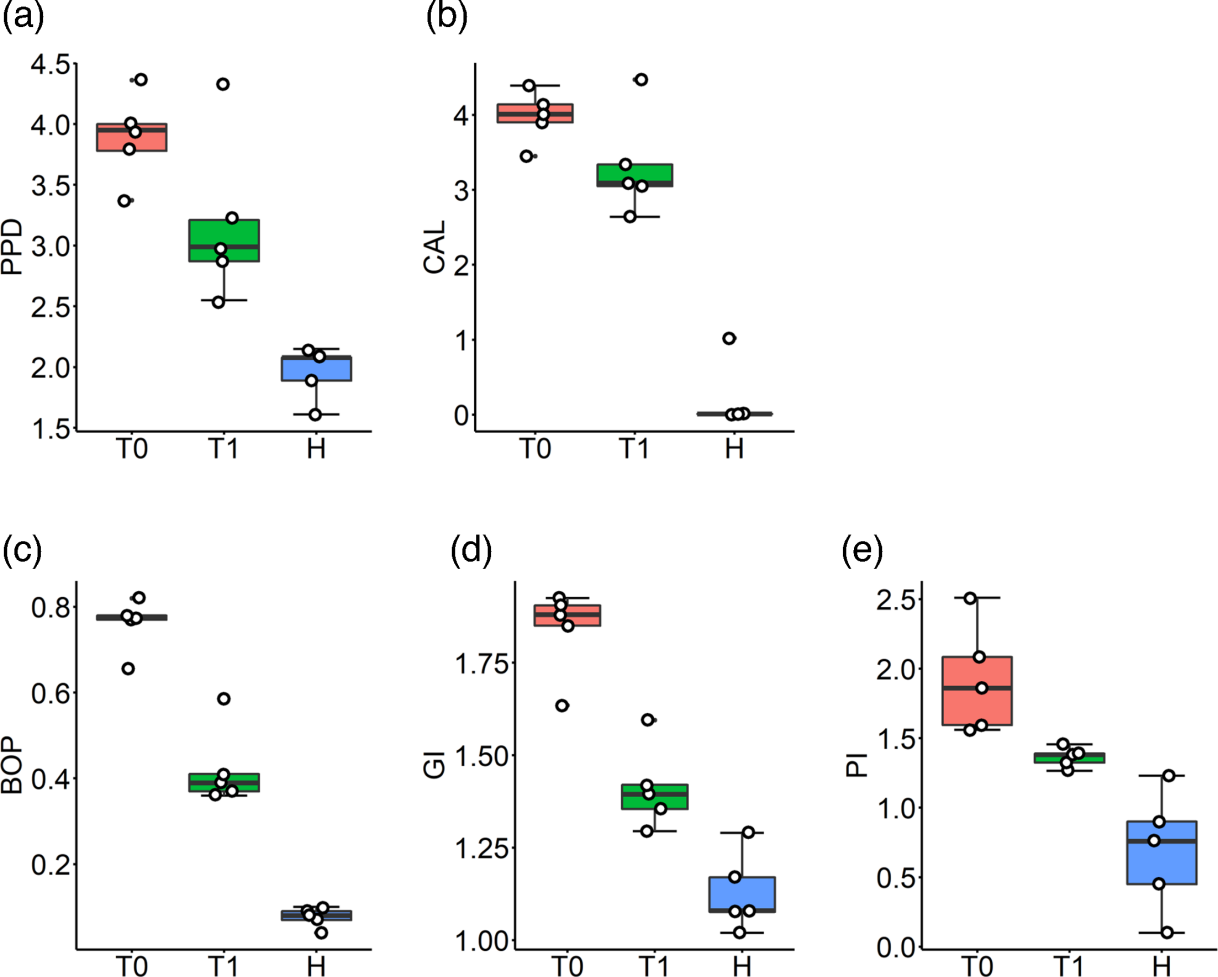
Clinical and periodontal parameters before (**T0**) and after 1 month (**T1**) non-surgical periodontal therapy of grade C periodontitis patients. H, healthy periodontium; PPD (mm), periodontal probing depth; CAL (mm), clinical attachment level; GI, gingival index; BOP, % bleeding on probing; PI, periodontal index.

The study was completed without any dropouts, and clinical improvement was observed in the clinical periodontal parameters after periodontal treatment in the grade C periodontitis group. There was a statistically significant difference between before (T0) and after treatment (T1) for BOP (*P*=9.10–6) (Fig. 1c) and GI (*P*=0.0001629) (Fig. 1d), while the difference between T0 and T1 was not significant for PD (*P*=0.076) (Fig. 1a), CAL (*P*=0.1021143) (Fig. 1b) and PI (*P*=0.0558758) (Fig. 1c). All clinical periodontal parameters: PPD, BOP, GI, PI and CAL in healthy controls (H) were significantly different from both T0 (*P*=0.00006, *P*=0.0e+00, *P*=0.0000010, *P*=0.0002493 and *P*=0.0000491) and T1 (*P*=0.00317, *P*=6.7e-06, *P*=0.0044906, *P*=0.0212992 and *P*=0.0016525) parameters of grade C periodontitis patients, respectively (Table 1 and Fig. 1)

### Composition of microbial community analysis

According to the taxonomic annotation results, the top ten taxa of each sample or group were selected at each taxonomic rank (phylum, class, order, family and genus) to form the distribution histogram of the relative abundance of taxa. When the microbial bacterial compositions were examined at the phylum level, it was determined that the saliva *Fusobacteriota* composition showed a significant decrease after NSPT (*P*<0.05). Besides gut composition, the decrease in *Firmicutes* with increased *Bacteroides* resulted in a decreased *Firmicutes*/*Bacteroides* ratio without statistical significance.

### Alpha diversity

**Fig. 2.**
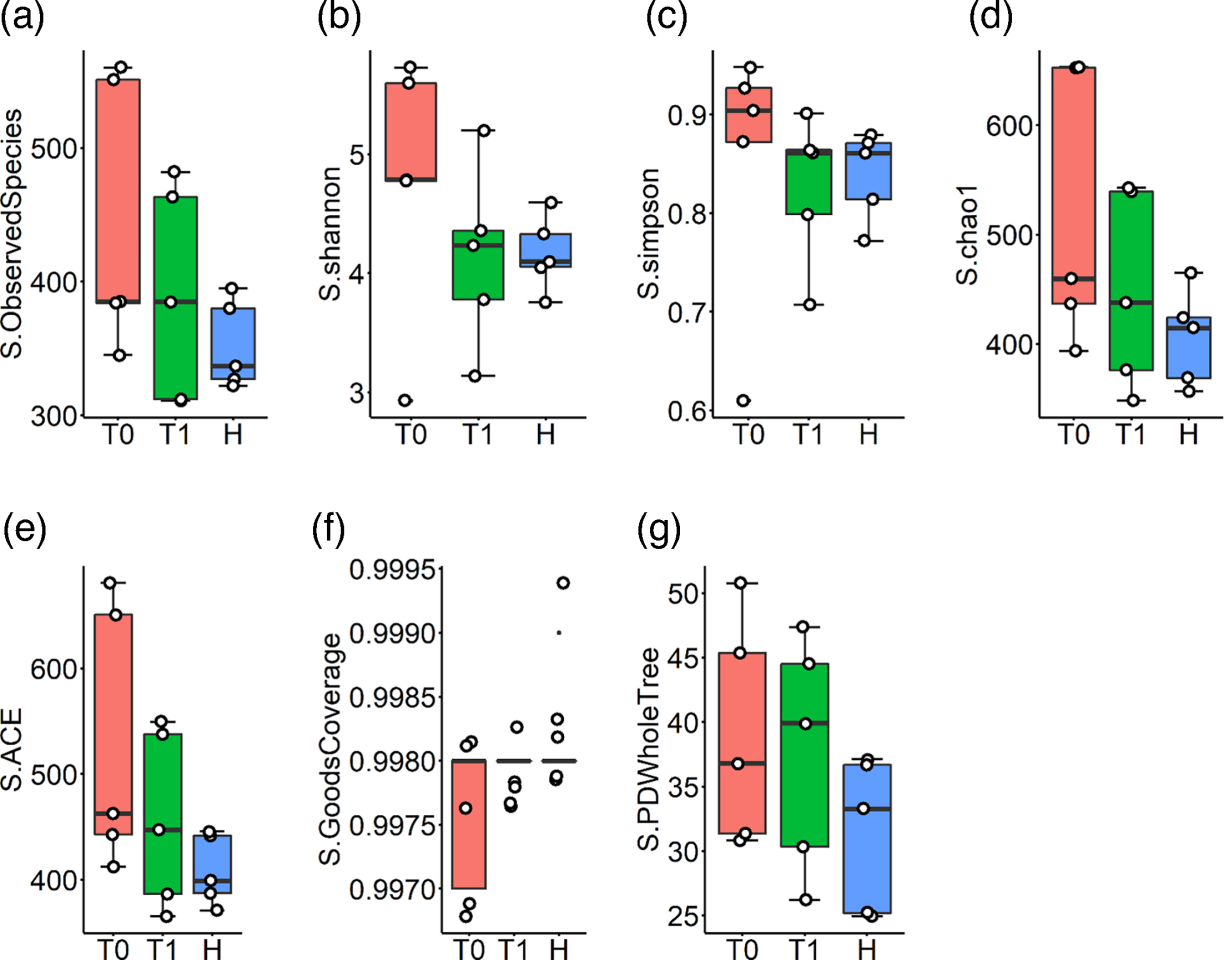
Salivary microbiota before (**T0**) and after 1 month (**T1**) non-surgical periodontal therapy of grade C periodontitis patients and healthy individuals. H, healthy periodontium; S, salivary microbiota.

**Fig. 3.**
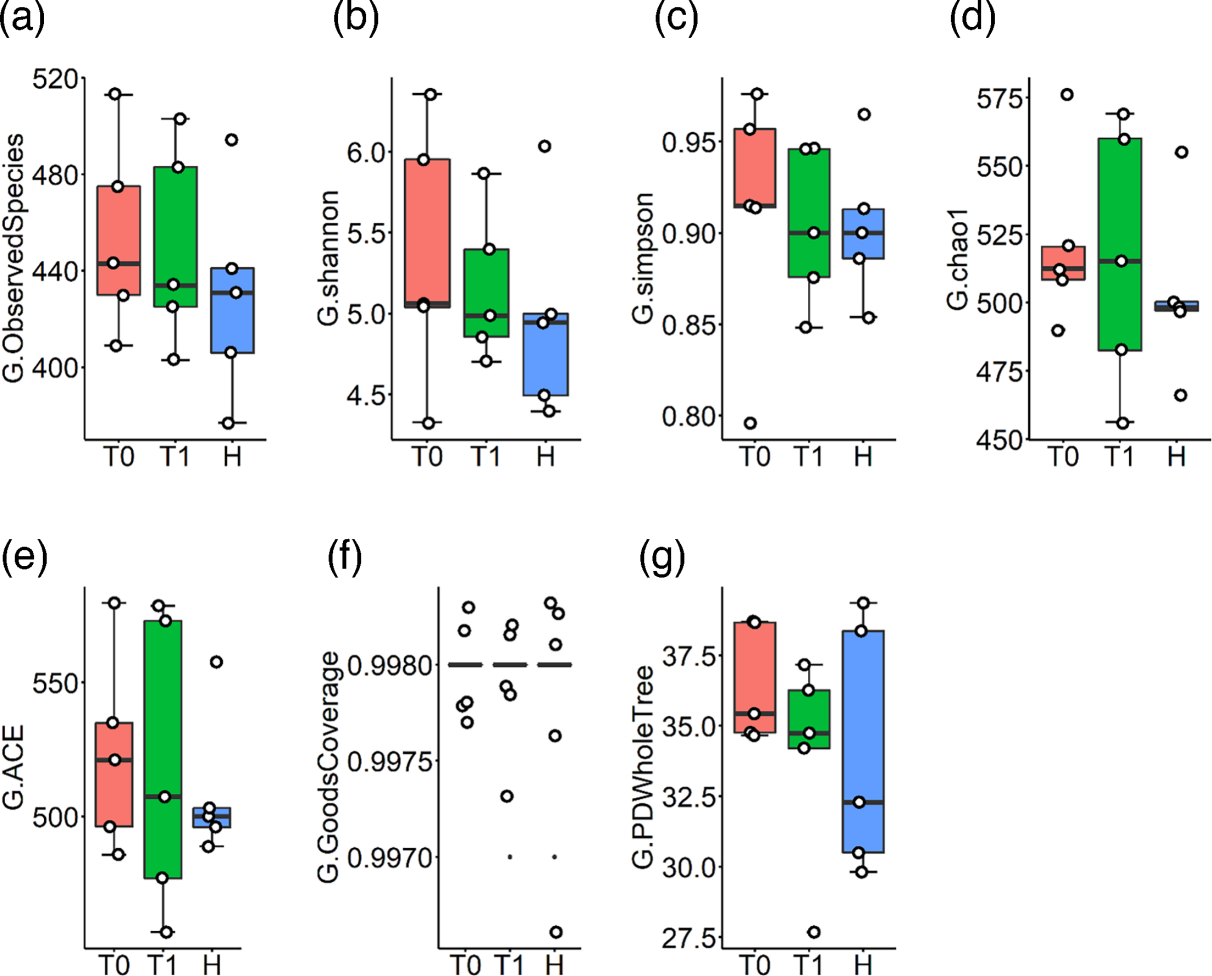
Gut microbiota before (**T0**) and after 1 month (**T1**) non-surgical periodontal therapy of grade C periodontitis patients and healthy individuals. H, healthy periodontium; G, gut microbiota.

Alpha diversity measures the microbial community diversity within a single sample, reflecting both the richness and diversity of microbial communities in each sample or group. In this study, alpha diversity was assessed to analyse the complexity of biodiversity in each sample and group using six indices: observed species, which counts the unique species present; Chao1, an estimator of species richness; Shannon, which accounts for both abundance and evenness of species; Simpson, which measures the probability that two randomly selected individuals belong to the same species; ACE, an abundance-based coverage estimator of species richness; and Good’s coverage, which evaluates the completeness of sampling. Although alpha diversity indices did not show a statistically significant change in T1, there was a trend in the saliva (Fig. 2) and gut (Fig. 3) microbiota profiles shifting towards those seen in healthy participants.

### Beta diversity

Beta diversity, a measure of the variation in microbial species composition between different samples or groups, was assessed using both weighted and unweighted UniFrac distances. This analysis allowed for the evaluation of group differences in microbial community complexity and composition, highlighting shifts in species diversity across the compared groups.

Contrary to saliva, the change in the beta diversity of gut microbiota after NPT was towards better health. Following NPT, the beta diversity of the gut microbiota shifted towards resembling the microbial profile of the healthy group, whereas the beta diversity of the salivary microbial profile did not show a distinct pattern.

### Correlation of microbiome alteration and periodontal parameters

**Fig. 4.**
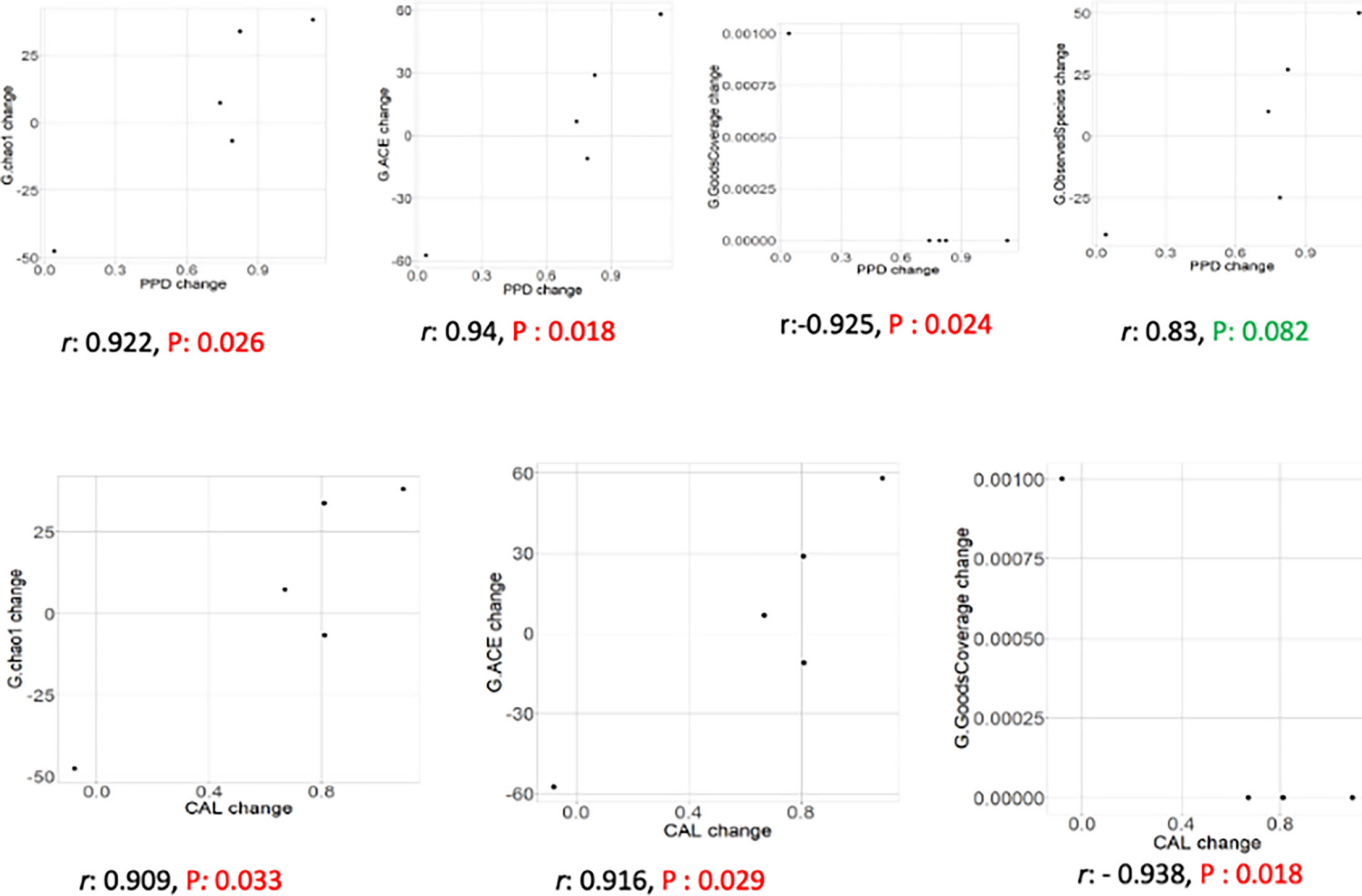
Correlation in the change of periodontal parameters (PPD and CAL) and gut microbiota in grade C periodontitis after treatment.

No correlation was found between the change in PPD, CAL, GI, BOP, PI and the change in salivary microbiota. While there was no correlation between GI, BOP and PI and gut microbiota, there is a correlation between change in PPD and CAL and change in gut microbiota in grade C periodontitis after treatment (Fig. 4).

## Discussion

The relationship between periodontal diseases and systemic inflammatory diseases and the contribution of non-surgical periodontal treatment to these systemic diseases and the overall health of the organism is supported by various literature studies. Immune host mechanisms are examined in detail to better elucidate chronic inflammatory disease pathogenesis. At the same time, microbiological communities and their interactions with different microenvironments (gut, oral, skin and vaginal) constitute a remarkable study area of microbiota studies. In addition to the local negative effects of dysbiosis, it has been determined that the dysbiosis of the gut microbiota presents remarkable interactions with systemic diseases and local effects. From this point of view, scientific studies that deal with treatment approaches that can provide a healthy microflora by treating dysbiosis, such as pre-probiotic approaches, are promising for overall health. In this study, besides its effect on oral health, the effects of non-surgical periodontal treatment on gut microbiota were evaluated, and it has been observed that the intestinal microbial composition of individuals with periodontal disease tends to change in a direction similar to the flora of healthy individuals after non-surgical periodontal treatment.

Microbial communities of the oral cavity are included in the intestinal microflora by a direct pathway through the gastrointestinal tract. In the presence of periodontal disease, deep periodontal pockets and increased plaque and calculus accumulation create an excellent reservoir for pathogenic bacteria, causing both local dysbiosis and the destruction of periodontal supportive tissue, and it is thought that it may affect the gut microbiota by advancing along the intestinal tract. It has been determined that a significant amount of oral bacteria reaches the intestine through swallowing saliva, food and beverages, and these oral pathogens colonize the region more intensely in gastrointestinal system diseases such as colon cancer, gastro-oesophageal reflux and inflammatory bowel disease [11]. It has been determined that *Porphyromonas gingivalis*, a potent periodontal pathogen, has a role in orodigestive cancers by colonizing the intestine by progressing along the gastrointestinal tract (GIS) with its acid resistance feature [12]. Studies report significant changes in mice’s gut microbiota after oral administration of high doses of *P. gingivalis*. Increased local and systemic inflammation, insulin resistance, systemic endotoxaemia and decreased intestinal barrier function were detected. While no significant changes were observed in the diversity analyses, an increase in *Bacteroidetes* and *Prevotella* and a decrease in *Firmicutes* are reported in gut microbiota [13]. Although the lack of detailed microbiological analyses of important keystone pathogens such as *P. gingivalis* and *Fusobacterium nucleatum* is a limitation of our study, the relative abundance of microbial communities reveals essential findings, especially at the phylum level. Compared to pretreatment distribution in the saliva microbial community after NSPT, a higher abundance of *Firmicutes* and a lower abundance of *Bacteroidetes*, *Spirochaetes* and *Fusobacteria* were detected.

Increased *Firmicutes*/*Bacteroides* ratio may be an indicator of gut dysbiosis, especially in obesity, and this ratio decreases with weight loss [14]. At the same time, an altered gut *Firmicutes*/*Bacteroides* ratio is related to various GIS diseases and inflammatory diseases such as lupus and diabetes [15]. In this study, the relative abundance ratio of two essential phyla of gut microbiota decreased after NSPT, with an increase in *Bacteroides* and a decrease in *Firmicutes*. Although the relationship between the saliva *Firmicutes*/*Bacteroides* ratio and oral dysbiosis/health is not established, it is known that *Firmicutes* is abundant over *Bacteroides* in healthy conditions, and *Bacteroides* is found in periodontitis-associated phyla [16]. According to the findings of this study, it is seen that the abundance of *Firmicutes* in saliva after NSPT was found to be higher. At the same time, with the decrease in the abundance of *Bacteroides*, a decreased *Firmicutes*/*Bacteroides* ratio occurs in saliva. It is observed that the relative abundance distributions of both gut and saliva seem more similar to healthy individuals in the periodontitis group after NSPT. The notable reduction in periodontopathogens as *Spirochaetes* and the *Fusobacteria* phylum, following NSPT is an additional sign of the transition towards a healthier saliva environment.

According to the results of metagenomic analysis, it was determined that the microbial profile after periodontal treatment showed a trend to change to be more similar to healthy individuals. At the same time, no statistically significant difference was found after NSPT in alpha diversity indices in gut and saliva (Figs2  3). However, it was observed that this change in microbial profile showed a strong correlation with the change in clinical periodontal parameters CAL and PPD (Fig. 4). It is thought that monitoring this correlation in PPD and CAL is crucial, which are subjective clinical parameters to evaluate periodontal treatment success. At the same time, it is thought that this finding can be explained by the fact that the deep periodontal pockets observed in the grade C group are an important reservoir affecting the microbial flora and the decrease in the pocket depths can be effective in the change of both oral and gut microflora (Table 1). Saliva samples were preferred as a diagnostic tool in this study. However, it is seen as another limitation that the effect of the decrease in pocket depths after periodontal treatment on microbial composition can be evaluated more precisely with subgingival plaque samples.

Along with the direct pathway in the relationship between the gut and oral microflora, in which bacteria are transmitted along the GIS tract, chronic systemic inflammation and host response constitute another important interaction pathway between the oral and gut microbiota [5]. While forming the study groups, the saliva and gut microbial compositions of periodontally healthy individuals and individuals with grade C periodontitis of a similar age were compared. In the clinical features of grade C periodontitis, progressive periodontal destruction is not proportional to age and the amount of microbial accumulation, which was defined as aggressive periodontitis in previous classifications [3]. However, the specific biochemical, immunological and/or genetic aetiology that distinguishes grade C or individuals with aggressive periodontitis from other periodontitis cases has not been fully elucidated, and it is accepted in the current classification that a disease is a severe form of a similar clinical picture. The absence of a statistically significant microbial profile pattern after periodontal treatment in the grade C periodontitis group can be explained by the different and complex immune response mechanisms that individuals may have. However, while clinical parameters indicate improvement in these individuals with severe periodontal destruction, the possibility of the disease not being fully controlled and the 1-month post-treatment period may be insufficient to talk about a complete recovery. Considering the need for a more extended period for the change in one of these complex pathways to reflect on the other, this pilot study might lead to further longitudinal studies, such as 3, 6 and 12 months after NPT. Studies in which individuals with different periodontal conditions, such as grade B periodontitis and gingivitis, periodontally healthy individuals and individuals with grade C periodontitis, are evaluated together may also provide more explanatory results.

## Conclusion

Metagenomic analysis methods provide detailed insights into the microbial compositions of various microenvironments, aiding in the understanding of host–microbiome interactions within these environments. In studying these interactions, omic approaches such as metabolomics and proteomics, which offer real-time functional data on microbiota activity, will further enhance our understanding of the impact of periodontal disease on the gut microbiota. These approaches, in conjunction with metagenomic analysis, will also shed light on the relationship between periodontal disease and systemic health.

## Supplementary material

10.1099/jmm.0.002065 Uncited Supplementary Material 1.

## Abbreviations

BMI: body mass index
BOP: bleeding on probing
CAL: clinical attachment level
GI: gingival index
NSPT: non-surgical periodontal treatment
PI: plaque index
PPD: periodontal pocket depth
