# Corrigendum: Correlation in the change of gut microbiota with clinical periodontal parameters in grade C periodontitis patients after non-surgical periodontal therapy

**DOI:** 10.1099/jmm.0.002120

**Published:** 2026-01-13

**Authors:** Elif Mutafcilar Velioglu, Uğur Arslan, Seyit Ali Kayis, Salih Maçin, Nobuhiko Kamada, Sema S. Hakki

**Affiliations:** 1 Department of Periodontology, Faculty of Dentistry, Selcuk University, Konya, Turkey; 2 Department of Microbiology, Faculty of Medicine, Selcuk University, Konya, Turkey; 3 Department of Biostatistics, Faculty of Medicine, Bolu Abant Izzet Baysal University, Bolu, Turkey; 4 Department of Internal Medicine, Division of Gastroenterology, University of Michigan Medical School, Ann Arbor, MI, USA

**Keywords:** gut microbiota, metagenomics, oral microbiota, periodontal health, periodontitis

In the published version of this article, there was a missing initial in the last author’s name.

The name should have appeared as ‘Sema S. Hakki’. This has now been corrected in the author list above and in the published article.

The authors apologise for any inconvenience caused.

