# Supplementary material for: Correlation in the change of gut microbiota with clinical periodontal parameters in grade C periodontitis patients after non-surgical periodontal therapy
Source: J Med Microbiol. 2025 Oct 9;74(10):002065. doi: 10.1099/jmm.0.002065 (PMC12510808; doi:10.1099/jmm.0.002065)
Supplement: Uncited Supplementary Material 1. [file jmm-74-02065-s001.pdf]

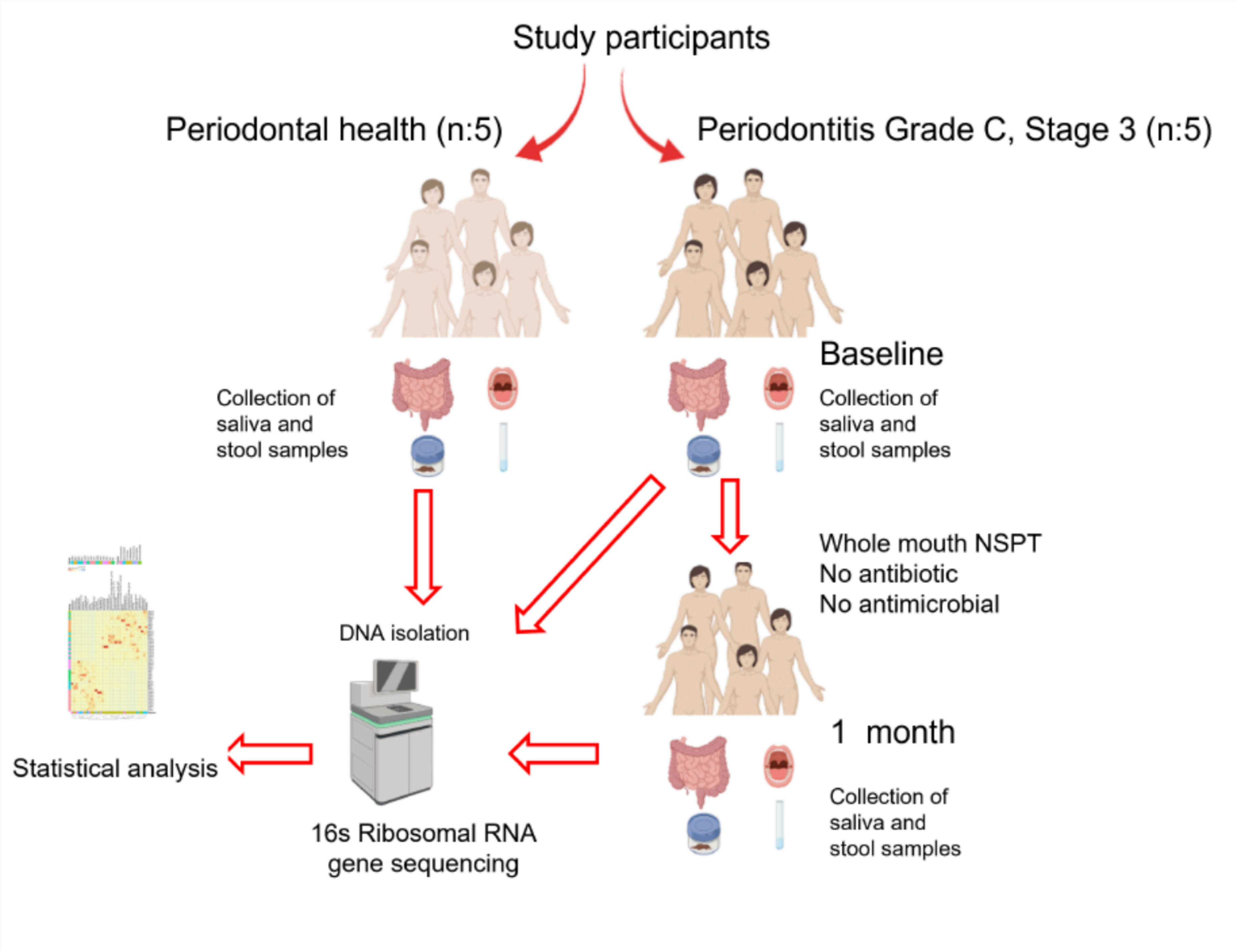

Supplementary Figure 1: Study design of the study. NSPT: Nonsurgical periodontal therapy

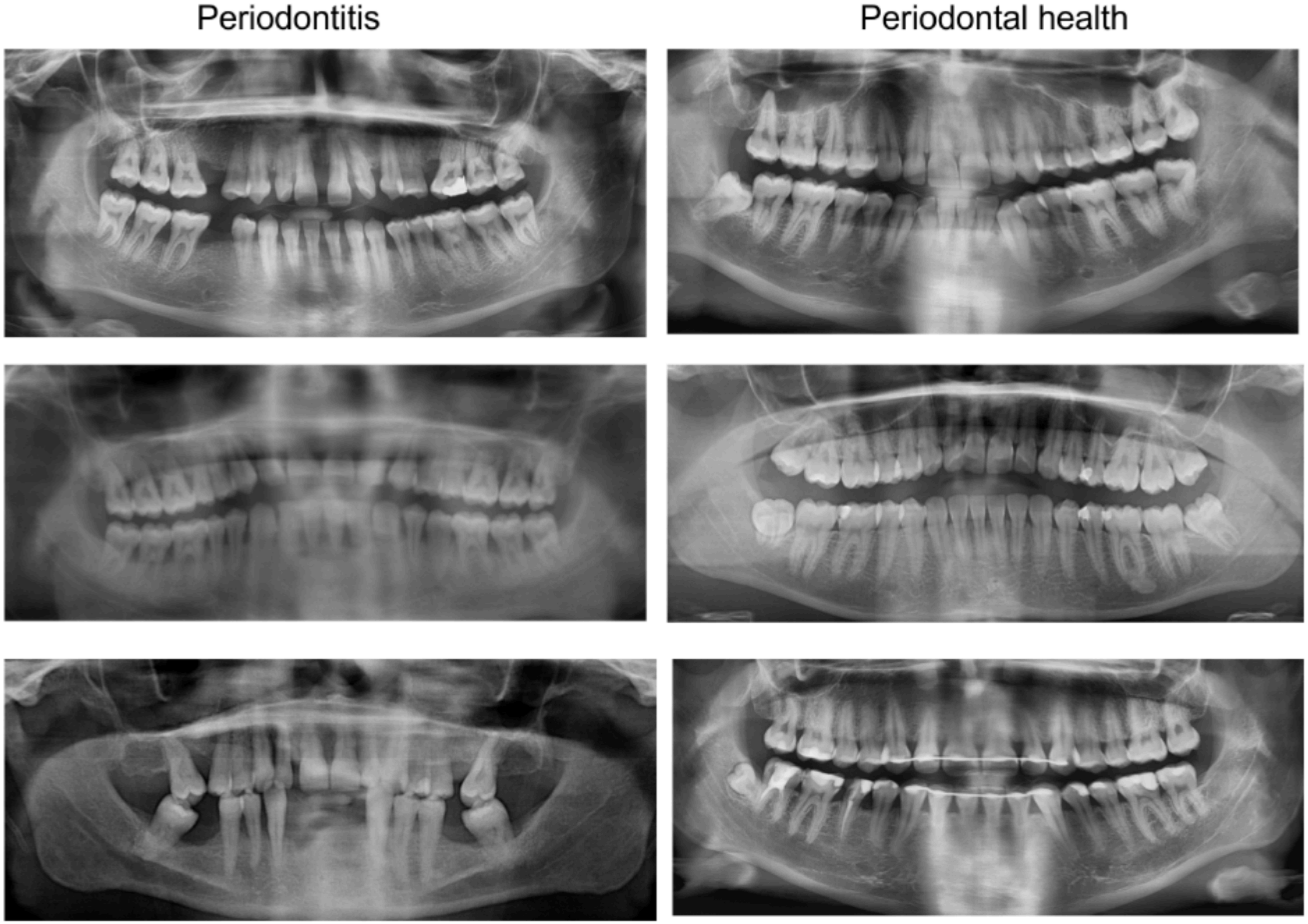

Supplementary Figure 2. Radiographic records of participants. Left column: periodontitis; right column: periodontal health.
